# Endometrial carcinosarcoma in a young female with absence of established risk factors: a case report

**DOI:** 10.3389/fonc.2025.1656841

**Published:** 2025-09-25

**Authors:** Na Wei, Shifeng Bai, Donghui Kong, Xiaomin Wang, Xin Mi

**Affiliations:** ^1^ Tangshan People’s Hospital, Tangshan, Hebei, China; ^2^ National Innovation Center for Advanced Medical Devices, Shenzhen, Guangdong, China; ^3^ Cancer Hospital of Dalian University of Technology, Shenyang, Liaoning, China; ^4^ Liaoning Cancer Hospital & Institute, Shenyang, Liaoning, China; ^5^ Shunyi Women’s & Children’s Hospital of Beijing Children’s Hospital, Beijing, China

**Keywords:** endometrial carcinosarcoma, young female, no established risk factors, multidisciplinary management, case report

## Abstract

Endometrial carcinosarcoma is a rare, aggressive uterine malignancy, usually affecting postmenopausal women with recognised risk factors such as prolonged unopposed oestrogen exposure, tamoxifen use, pelvic irradiation, or high BMI. We report a 21-year-old Chinese woman with no known risk factors who presented with persistent vaginal bleeding, anaemia, and lower abdominal pain. Investigations showed microcytic hypochromic anaemia, elevated CA125, CA153, CA199, and Human Epididymis Protein 4, and pelvic MRI revealed a malignant endometrial lesion with vaginal involvement and lymphadenopathy. Biopsy confirmed carcinosarcoma with grade 3 endometrioid carcinoma as the epithelial component. Multidisciplinary review recommended extensive laparoscopic hysterectomy with bilateral adnexectomy, lymphadenectomy, and omentectomy, followed by six cycles of paclitaxel–carboplatin chemotherapy. Histology confirmed the diagnosis, and two-year follow-up showed no recurrence; a postoperative vesicovaginal fistula was surgically repaired. This atypical case in a young, underweight woman without established risk factors challenges current epidemiological assumptions and emphasises the importance of broad diagnostic consideration, timely referral, and comprehensive management.

## Introduction

Endometrial carcinosarcoma, also known as Malignant Mixed Müllerian Tumour (MMMT), is a rare and aggressive form of endometrial cancer. It originates in the endometrium, the lining of the uterus, and is characterized by the presence of both carcinomatous (epithelial) and sarcomatous (mesenchymal) components within the tumour. This dual nature makes endometrial carcinosarcoma a unique and complex malignancy, with features of both endometrial carcinoma and uterine sarcoma.

Endometrial carcinosarcoma accounts for approximately 5% of all endometrial cancers (1). It predominantly affects postmenopausal women, with the majority of cases occurring in women average aged 67 (1). The exact cause of endometrial carcinosarcoma remains unclear, but several risk factors have been identified, including age, high BMI, oestrogen-only hormone replacement therapy without progesterone, tamoxifen use, and prior pelvic radiation therapy (2).

In this report, we present a unique case of endometrial carcinosarcoma in a young Chinese patient who was admitted without any known risk factors.

## Case presentation

A 21-year-old female was admitted to hospital with persistent vaginal bleeding for over six months and recent lower abdominal pain lasting for more than two weeks. At the onset of the bleeding six months earlier, she was diagnosed with mild anaemia at a local clinic. Treatment with cephalosporins and intravenous anti-inflammatory medications was initiated; however, after eight days, her condition remained unchanged, but no further medical interventions were sought. Two months prior to her hospital admission, she experienced fatigue and visited the local clinic again. A reassessment indicated moderate anaemia, for which she was prescribed ‘Blood Enrichment Mixture ‘ and iron supplements. After taking the medication, her fatigue symptoms improved, but she did not undergo further examinations or receive additional treatment. Approximately two weeks before her admission, she began experiencing lower abdominal discomfort and persistent dull pain. She sought medical assistance at a local clinic, where she was prescribed anti-inflammatory medications. After eight days of medication use, she experienced a mild improvement in her lower abdominal pain; however, no further examinations or treatments were conducted. Two days before her admission, she developed severe pain in the right lower abdomen. Self-administered painkillers provided minimal relief, prompting her to seek further medical attention.

**Figure 1:**
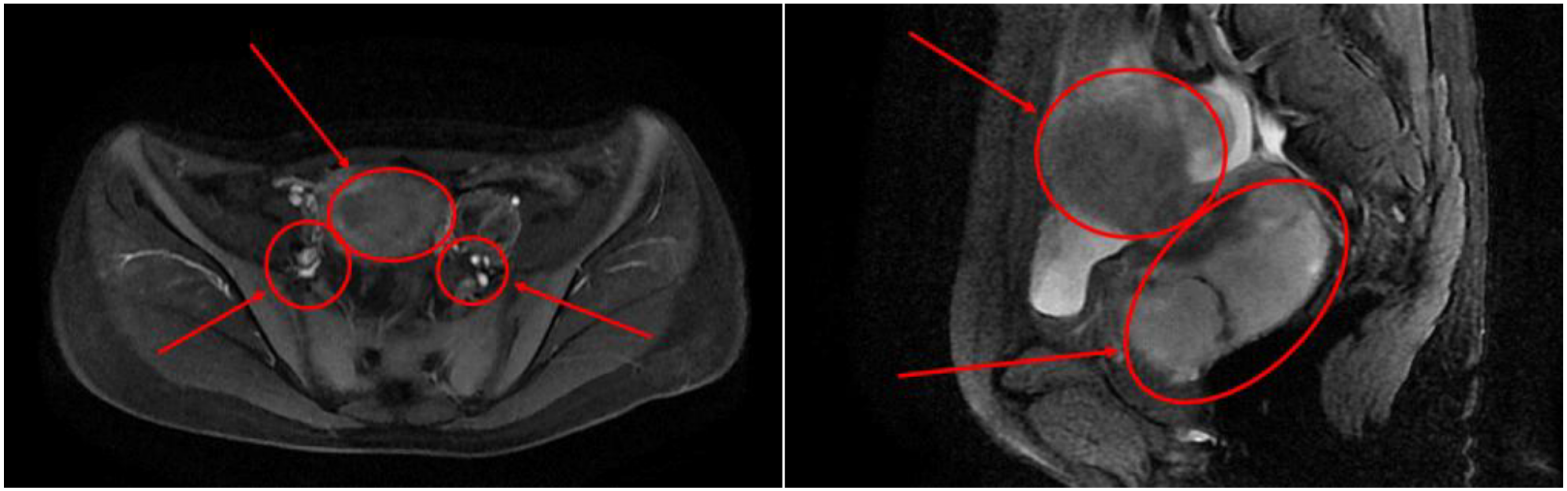
Cross-sectional pelvic MRI and sagittal pelvic MRI findings.

**Figure 2:**
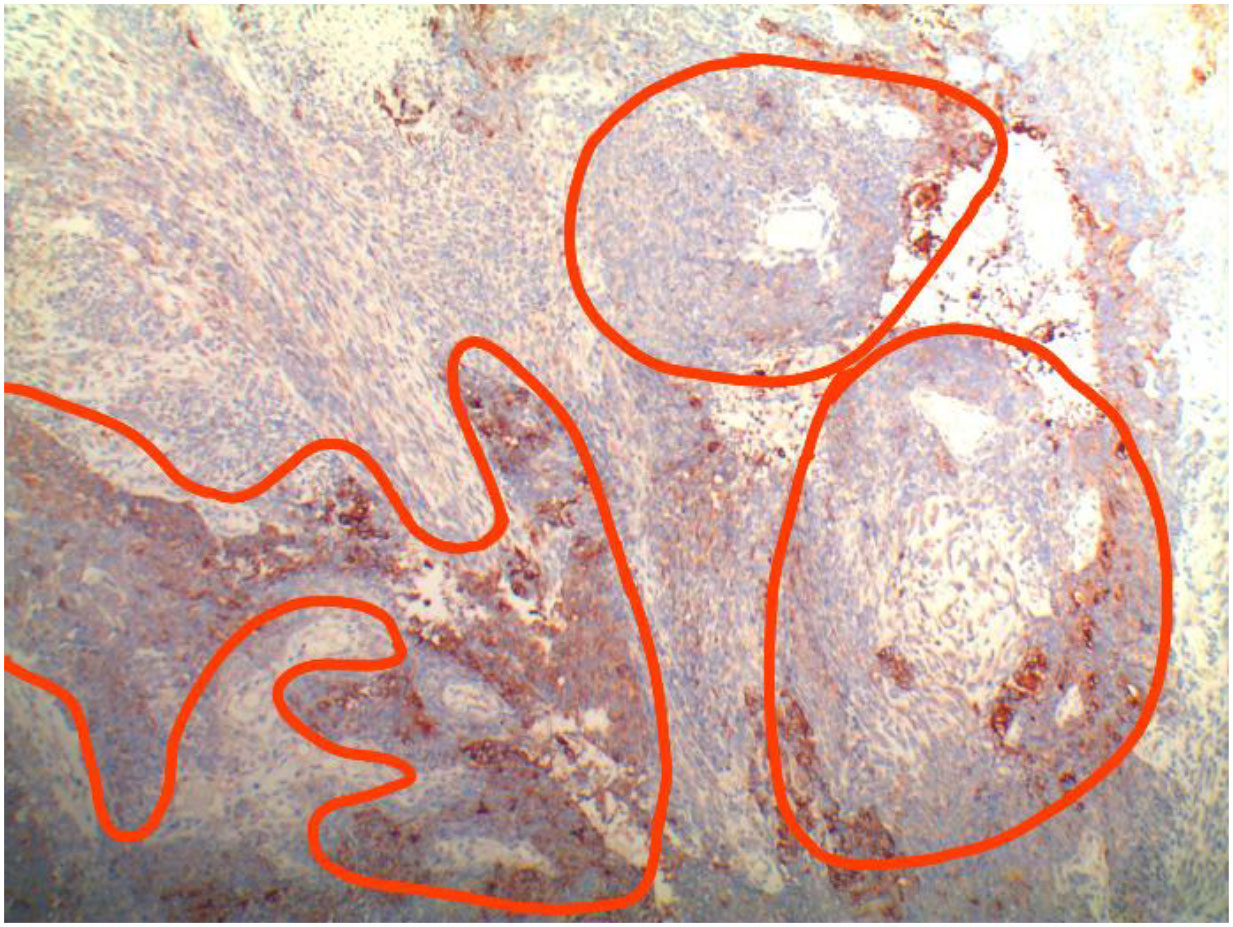
Histopathological findings of the vaginal mass biopsy.

After admission, the patient was found to have tachycardia with a heart rate of 127 beats per minute. Blood cell analysis indicated an elevated white blood cell count of 12.44 x 10^9/L, with an increased percentage of neutrophils at 84.4%. Haemoglobin levels at 82g/L, haematocrit at 31.70%, mean red blood cell volume of 74.4fL, and a platelet count of 729 x 10^9/L confirmed the presence of microcytic hypochromic iron-deficiency anaemia. Stool analysis suggested the positive presence of occult blood. Tumour marker tests for females revealed levels of CA-125 at 117.30U/mL, CA-153 at 46.04U/mL, CA-199 at 74.47U/mL, and Human Epididymis Protein 4 at 572.80pmol/L. Pelvic MRI showed abnormal uterine morphology and signals as shown in **Figure 1**, suggesting a malignant tumour that had affected the middle and lower parts of the vagina, accompanied by multiple enlarged lymph nodes on both sides of the pelvic wall. As the patient reported no history of sexual activity, a biopsy of the vaginal mass was performed only after the MRI suggested the presence of a malignant tumour. Biopsy histopathological analysis confirmed a diagnosis of carcinosarcoma, with the epithelial component identified as grade 3 endometrioid carcinoma, as shown in **Figure 2**.

The Multidisciplinary Team (MDT) conducted a comprehensive review of the patient’s MRI, CT, ultrasound and pathological findings. Above studies indicated abnormal uterine morphology and signal changes suggestive of a malignant tumour, with involvement of the middle and lower vaginal segments, as well as multiple enlarged lymph nodes on both sides of the pelvic wall. Given the potentially advanced stage of the disease and the presence of lymphadenopathy, fertility-sparing surgery was deemed unsuitable.

In accordance with the Chinese Society of Clinical Oncology (CSCO) guidelines 2022, the MDT recommended surgical intervention, with the primary objective of achieving maximal cytoreduction. The proposed surgical approach included a laparoscopic extensive total hysterectomy with bilateral adnexectomy, pelvic lymphadenectomy, para-aortic lymphadenectomy, omentectomy, and lysis of intestinal adhesions. Postoperatively, adjuvant chemotherapy with paclitaxel and carboplatin was planned, alongside close monitoring to allow for an individualised treatment strategy based on the patient’s response.

The patient and her family were fully informed of the treatment plan and provided their consent. According to clinical guidelines, genetic testing is recommended at the time of histopathological confirmation of endometrial carcinoma. However, due to financial constraints and the lack of insurance coverage for this test, genetic testing was not performed.

On February 3rd, 2023, a surgical procedure was performed according to MDT’s suggestion. Intraoperatively, a small volume of ascites was noted, and a single markedly enlarged pelvic lymph node was identified in the left pelvis measuring 4.5 cm. Together, these findings were consistent with advanced disease and possible nodal metastasis. The patient had no history of previous surgeries, and there were no signs of past abdominal incisions. However, during the operation, it was observed that the intestines were tightly adherent to the pelvic wall, encasing the uterus, which was barely visible.

**Figure 3:**
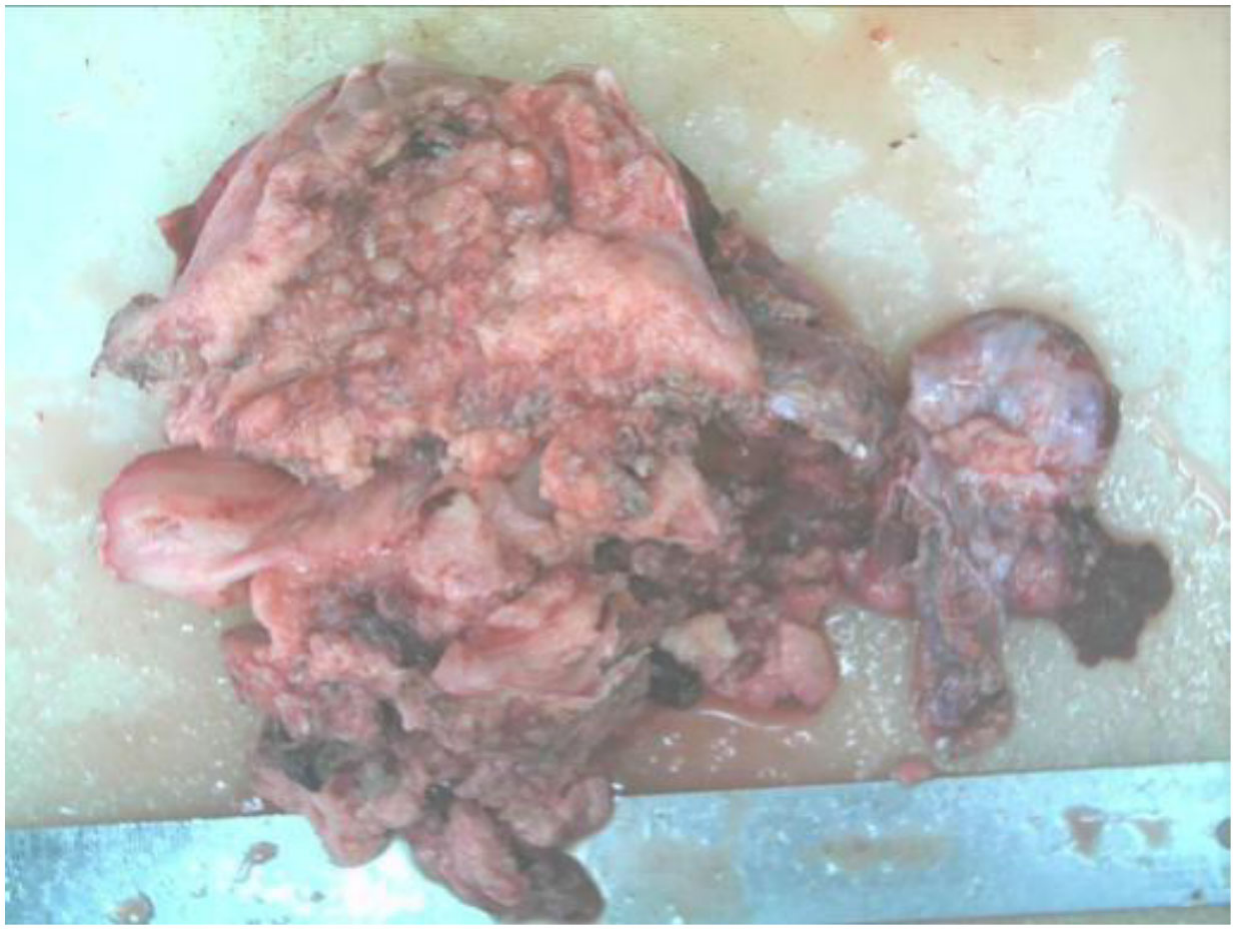
Resected carcinoma specimen.

**Figure 4:**
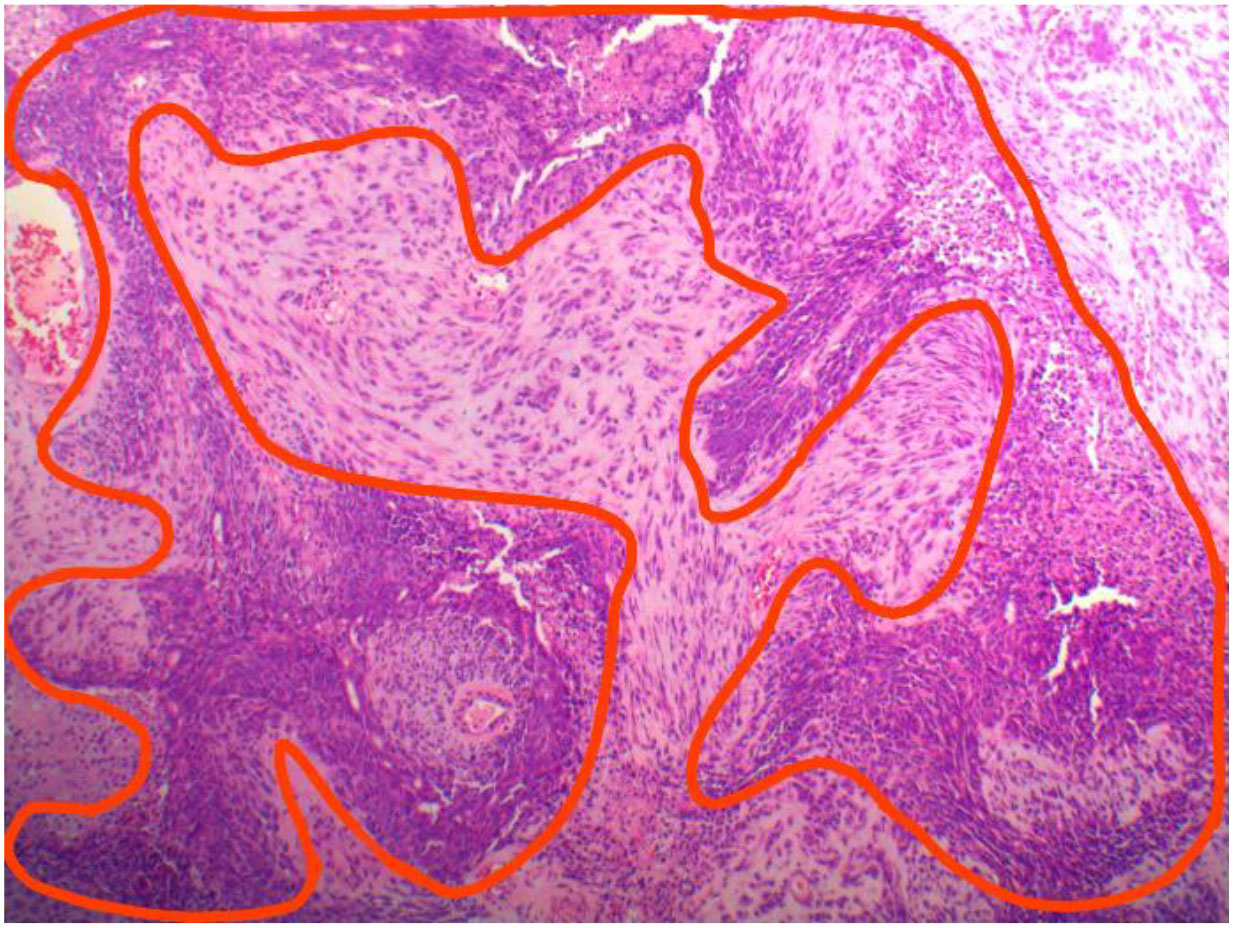
Postoperative histopathology of carcinosarcoma: carcinomatouscomponent outlined in red and sarcomatous component outside.

The postoperative pathological report diagnosed the specimen as carcinosarcoma with its epithelial component being grade 3 endometrial-like carcinoma as shown in **Figures 3**, **4**.

**Figure 5:**
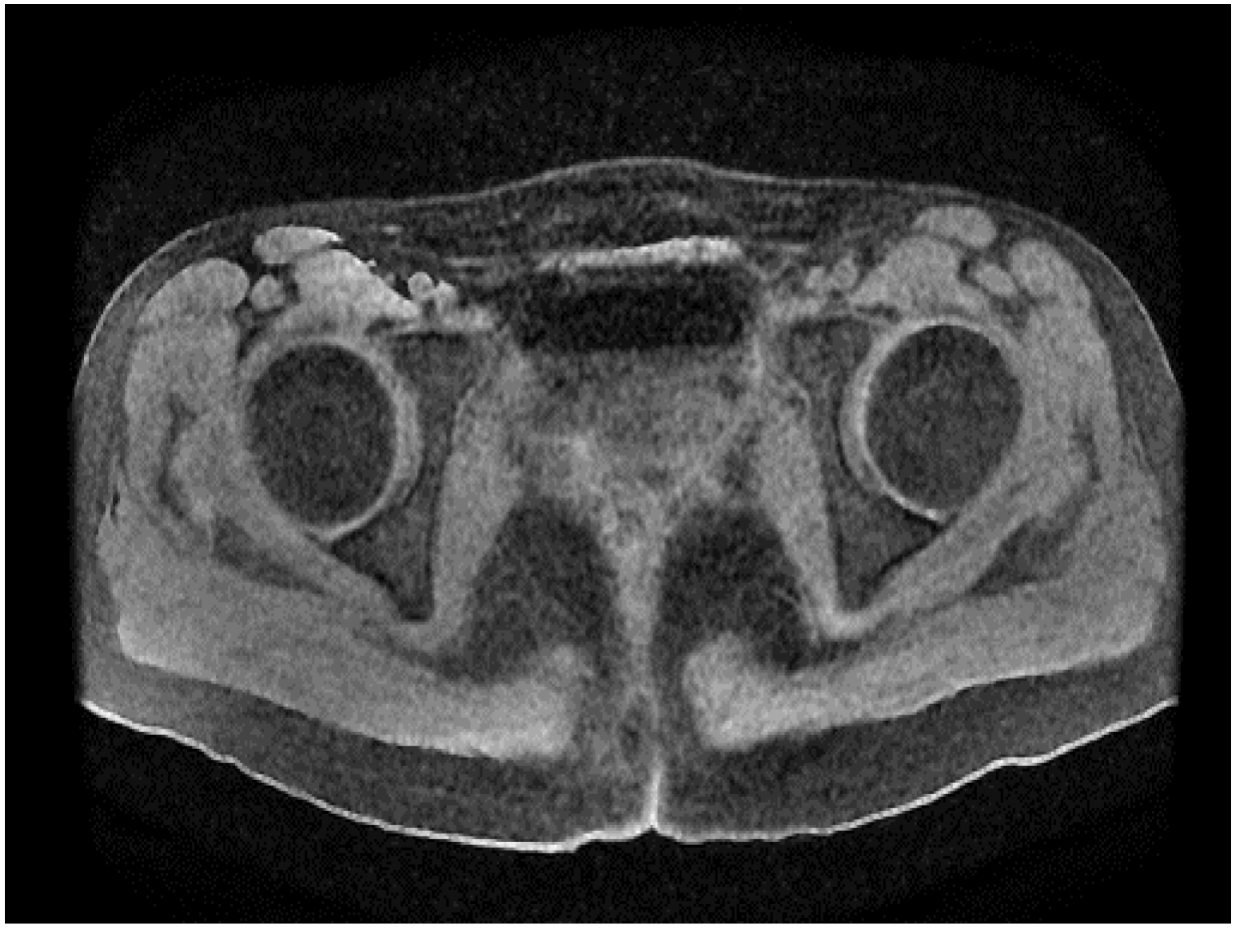
Pelvic MRI findings following six cycles of adjuvant chemotherapy.

Following surgery, the patient received adjuvant combination chemotherapy with paclitaxel and carboplatin. After completing six cycles, magnetic resonance imaging (MRI), as illustrated in **Figure 5**, showed no evidence of tumour recurrence or metastatic lesions. The tumour marker CA125 decreased significantly from a preoperative level of 117.3 to 11.63 prior to chemotherapy and subsequently remained within the normal range. CA153 levels also demonstrated a declining trend and remained within the normal range in the most recent evaluation. However, although the CA199 level decreased by over half postoperatively, from 77.47 to 30.74, it consistently remained above the normal threshold of 30 during follow-up assessments. As a result, the patient has been placed under regular monitoring by her local oncologist.

During the first postoperative year, follow-up assessments were undertaken at three-monthly intervals and comprised a general symptom inquiry, gynaecological examination, full blood count, pelvic ultrasonography, and contrast-enhanced computed tomography of the upper and lower abdomen and pelvis; a pelvic magnetic resonance imaging scan was additionally performed every six months. From the second year onward, the identical surveillance protocol has been maintained, albeit at six-monthly intervals. At each visit the patient reported no subjective discomfort, inspection of the vaginal vault revealed no palpable or visual evidence of residual or recurrent tumour, the full blood count remained unremarkable, and the serum tumour-marker panel stayed within reference limits. Serial pelvic ultrasound and magnetic resonance imaging demonstrated no structural abnormality, while the thoraco-abdominopelvic CT scans likewise showed no focal lesion or metastatic deposit.

Postoperatively, the patient developed a vesicovaginal fistula, which required surgical repair one year later. Following the repair procedure, the patient exhibited good spontaneous urination function, with no subsequent leakage from the bladder or vagina.

## Discussion

Endometrial carcinosarcoma is distinguished by the presence of both malignant epithelial and mesenchymal components. It constitutes roughly 5% of all uterine malignancies (1). As per the “Diagnose and Treatment Guideline for Endometrial Carcinoma Version 2022” (3), the prevalence of endometrial cancer in China is 10.28/100k people, accounting for 3.88% of female malignant tumours.

Numerous investigations (2) (4), have sought to identify risk factors linked to endometrial carcinosarcoma, with extended oestrogen exposure (5), previous pelvic radiation (6), and tamoxifen use (7) emerging as significant contributors. Yet, the correlation between BMI and this particular malignancy remains enigmatic. It’s widely acknowledged in oncological studies that a high BMI correlates with increased susceptibility to several cancers (8), endometrial carcinosarcoma included. Nevertheless, diagnosing a young patient with a low BMI with endometrial carcinosarcoma questions established beliefs, urging deeper probes into potential genetic, environmental, or yet-to-be-identified risk agents.

This case is particularly notable as it involves a young woman with a low body mass index (BMI), representing a marked deviation from the typical demographic profile of patients diagnosed with endometrial carcinosarcoma (1). The atypical nature of this presentation highlights the importance of maintaining a broad differential diagnosis, even among younger individuals generally considered to be at low risk. From a risk factor standpoint, the patient does not exhibit any of the commonly associated conditions linked to endometrial carcinosarcoma. She reports no history of sexual activity and has never used an intrauterine device. With a BMI of 17.9, she is categorised as underweight. Her menstrual cycles have remained regular, and there have been no reported abnormalities in diet or physical activity. Additionally, the patient has no history of diabetes, nor is there any personal or immediate family history of malignancy. The absence of known risk factors in this case underscores the need for further investigation into the aetiology and potential risk factors associated with this rare malignancy.

This report includes comprehensive imaging–pathology concordance, multidisciplinary decision-making, adherence to contemporary surgical and adjuvant standards, and systematic surveillance, thereby providing a complete care pathway for an exceptionally young patient. Limitations are inherent to a single-case design, the absence of molecular profiling (e.g., MMR/MSI, POLE, p53 status, HER2 assessment or broader next-generation sequencing), and a follow-up period that, while reassuring, remains relatively short for confidently excluding late recurrences. A postoperative vesicovaginal fistula was repaired, but quality-of-life impact was not formally assessed, and the persistent mild elevation of CA19–9 warrants continued monitoring without clear current clinical implications.

Beyond standard imaging and histopathology, additional decision-support tools could be used to refine pre-operative risk stratification and peri-operative planning. Pre-operative MRI-radiomics analyses (9) in endometrial carcinoma have shown promise in predicting tumour grade, deep myometrial invasion, lymphovascular space invasion (LVSI) and nodal metastasis—determinants that directly influence the extent of pelvic/para-aortic lymphadenectomy and the selection of adjuvant therapy. Because LVSI materially elevates recurrence risk yet cannot be reliably identified on routine imaging, radiomics-derived surrogates may help triage candidates for more extensive nodal assessment. Peri-operative risk estimation can likewise be strengthened by the modified fragility index (10), an established predictor of overall and serious post-operative complications, thereby informing prehabilitation, anaesthetic planning and enhanced-recovery pathways in high-risk histologies such as carcinosarcoma.

Reflecting on China’s status as a developing country, primary healthcare disparities remain particularly pronounced in remote rural regions (11). It is essential to acknowledge that, while common cancers are well-documented, rarer malignancies such as endometrial carcinosarcoma are often underrepresented in medical literature and clinical training. This contributes to an informational gap regarding awareness, diagnostic approaches, epidemiology, aetiology, and evidence-based treatment strategies, particularly within the Chinese population. Additionally, the vast expanse of China introduces variability in medical facilities, patient awareness, and clinical approaches throughout its regions. These differences heighten the complexity of detecting and adeptly addressing rare cancers.

In the future, strengthening collaborative ties between community clinics, regional health centres, cancer research organizations, and top-tier urban hospitals becomes crucial in honing diagnostic, treatment, and research endeavours. Apart from that, integrate molecular profiling (MMR/MSI, POLE, p53, HER2 and broader genomic panels) could be taken into consideration to refine risk stratification and identify candidates for targeted or immunotherapies, and systematically capture patient-reported outcomes including the impact of postoperative complications.

## Conclusion

In conclusion, this case of endometrial carcinosarcoma diagnosed in a young patient with no established risk factors underscores the possibility that this tumour can develop outside its typical demographic profile. The absence of high BMI, prolonged estrogen exposure, tamoxifen use, or prior pelvic radiation in our patient highlights the importance of maintaining clinical vigilance and an inclusive diagnostic perspective for gynaecological malignancies—even among those deemed at lower risk. Clinicians and researchers should consider the potential influence of unidentified genetic or environmental factors in such atypical presentations, particularly given that established risk factors do not universally apply.

Furthermore, this case illuminates the challenges posed by disparities in healthcare resources and awareness, particularly in rural or underserved regions. System-level collaboration is essential to improve timely diagnosis and care. By enhancing access to diagnostic tools, treatment protocols, and specialised training, the global medical community can work toward mitigating the adverse outcomes associated with endometrial carcinosarcoma, especially in younger patients without known risk factors.

## Data Availability

The original contributions presented in the study are included in the article/supplementary material. Further inquiries can be directed to the corresponding author.
